# Exposure Under Pressure: Lead Linked to Release of Cortisol in Children

**Published:** 2008-02

**Authors:** Julia R. Barrett

Lead exposure is linked to cognitive deficits, cardiovascular disease risk, and behavioral problems, outcomes that potentially follow dysregulation of the hypothalamic–pituitary–adrenal (HPA) axis. In animal studies, lead exposure has heightened the release of corticosterone, the counterpart to the human stress hormone cortisol. New research now reveals for the first time a similar response in children with blood lead levels below 10 μg/dL, the action level established by the Centers for Disease Control and Prevention **[*EHP* 116:249–255; Gump et al.]**. This finding corroborates concerns that there is no safe level of lead exposure.

The researchers drew their study population from the ongoing Oswego Children’s Study, a longitudinal study at the State University of New York at Oswego’s Center for Neurobehavioral Effects of Environmental Toxics. Of the 169 children in the current study, blood lead levels were known for 154 prenatally (≤1–6.3 μg/dL) and for 120 during infancy or toddlerhood (1.5–13.1 μg/dL). At the time of their participation in the current study, children were 9.5 years old.

Cortisol levels vary diurnally, rising quickly after awakening and then declining steadily thereafter. To help control for this diurnal variation, tests always occurred in the late afternoon. Beginning with a brief rest period, each child’s session involved submerging an arm in ice water for 1 minute and completing a series of simple tasks with intervening rest periods—a standard protocol to assess neuro-endocrine response to acute stress. Saliva was collected for cortisol measurements during the first rest period and at 21, 40, and 60 minutes after the cold stressor test.

The researchers controlled for numerous potentially confounding factors, including demographics, socioeconomic status, and the health, nutrition, and substance use of mothers and children. They also tested for the presence of other neurotoxicants such as poly-chlorinated biphenyls, DDE, and hexachlorobenzene in children’s blood, as well as maternal mercury exposure.

Pre- and postnatal blood lead were not associated with any variation in baseline cortisol levels. However, increasing blood lead levels were independently and significantly associated with increasing cortisol responses to stress. Curiously, cortisol levels remained elevated throughout the test period instead of tapering off as expected. The authors suggest that the children may have already been stressed when the test began or that 60 minutes was insufficient for cortisol levels to return to baseline.

The precise mechanisms of lead’s effect on the HPA axis are unclear. However, given the effects they found at relatively low lead exposures, the authors suggest that cortisol reactivity be considered in future studies as a potential mediator of lead-induced disorders.

